# Intraprocedural cortisol testing improves adrenal vein cannulation success and diagnostic accuracy in assessment of primary aldosteronism, in a medium throughput centre

**DOI:** 10.1038/s41371-022-00756-z

**Published:** 2022-09-30

**Authors:** Mahesh M. Umapathysivam, Bethany Morgan, Carmen Bischoff, Annabelle Hayes, Michael Wilks, Michael Stowasser, David J. Torpy

**Affiliations:** 1 Central Adelaide Local Health Network, SA Health, Adelaide, SA Australia; 2 University of Adelaide, Adelaide, SA Australia; 3 University of Queensland, Brisbane, QLD Australia

**Keywords:** Adrenal gland diseases, Diagnosis

## Abstract

Primary aldosteronism is the most common cause of secondary hypertension. Identifying individuals who have unilateral secretion from aldosterone secreting adenomas allows adrenalectomy. Surgical treatment when feasible may be superior to medical management with improved cardiovascular outcomes and reduced medication dependence. Adrenal vein sampling (AVS) is required to biochemically lateralise aldosterone secretion prior to adrenalectomy. However, diagnostic success of AVS is variable and can be poor even at tertiary centres; failure is largely due to unsuccessful adrenal vein cannulation. Intra-procedural rapid semiquantitative cortisol testing (RCT) identifies correct catheter placement in real time. We compared diagnostic success rates of AVS before and after the introduction of intraprocedural cortisol testing at the Royal Adelaide Hospital—a medium throughput tertiary centre (average 6.2 procedures a year over the last 8 years). We observed an increase in success rate from 63% to 94%. Intraprocedural cortisol testing also led to a net financial saving of ~$100 AUD per procedure. RCT is likely to be cost effective if pre-RCT success rate is less than 78%. Procedure time and number of samples collected, however, were increased with RCT. This suggests that intraprocedural cortisol testing will improve success in low to medium throughput centres and may make AVS feasible in less specialised centres.

## Introduction

Primary aldosteronism (PA) is the most common cause of secondary hypertension [1–3]. Independent of blood pressure effects, PA is associated with increased rates of stroke (OR 2.6), atrial fibrillation (OR 3.5), coronary artery disease (OR 1.8), heart failure (OR 2.1) and metabolic syndrome (OR 1.5) [4, 5]. Treatment directed at reducing aldosterone action reduces the risk of these events [6, 7]. In unilateral disease, which comprises 30–50% of PA, surgical adrenalectomy is the preferred treatment and results in cure of hypertension, medication independence in >50% of individuals and almost universal cure of hypokalaemia [8]. Additionally, surgical management when compared to medical management is associated with lower rates of atrial fibrillation, heart failure, stroke, and incidence of chronic kidney disease [6, 7, 9]. To proceed safely to adrenalectomy, adrenal vein sampling is required to localise aldosterone secretion and is the gold standard for lateralisation. This is true even in the presence of a unilateral adrenal lesions identified on adrenal imaging, except, perhaps in young individuals. Confirmation is important due to the relative high frequency of biochemical discordance with imaging, which has been reported to be as high as 46% [10]. Additionally, removal of the wrong gland results in the exposure to risk of an invasive adrenalectomy with no benefit and precludes removal of the contralateral gland due to the risk of adrenal insufficiency.

Adrenal vein sampling is technically difficult, expensive and many centres have a high failure rate (Supplementary Table 1), in one national referral centre the success rate was only 41%. This is largely attributable to the complex anatomy of the right adrenal and difficulty catheterising the right adrenal vein which radiographically resembles the right hepatic accessory vein and drains directly into the inferior vena cava.

We introduced intraprocedural semiquantitative rapid testing for cortisol in cannulated veins considered likely to be adrenal to guide judgement of AVS procedure success in our medium frequency centre (average of 6 procedure a year over the study period). This study has examined our success rates of the AVS procedure before and after the rapid testing and considered the procedure’s cost effectiveness.

Administration of ACTH during AVS stimulates cortisol secretion by the adrenal glands resulting in higher concentrations of cortisol in the adrenal veins which declines as the adrenal vein joins other veins until it progressively reduces to the peripheral plasma concentration. Measurement of cortisol concurrently with aldosterone allows identification of catheter placement by comparing cortisol concentration in the adrenal vein to peripheral cortisol concentration and is used to account for potential dilution of adrenal vein blood with partial cannulation [11]. A ratio of 3:1 between central to peripheral cortisol is a requirement for a diagnostic test but this is typically greater than 5:1 under ACTH stimulation [8]. Real time measurement using commercially available point of care rapid semi-quantitative cortisol testing (RCT) to identify plasma with low level cortisol 276–860 nmol/L (expected peripheral cortisol concentration) and plasma cortisol concentration >820 nmol/L (expected adrenal vein cortisol concentration) will allow differentiation of the blood from the adrenal vs non-adrenal veins [12]. This technique has been utilised to improved success rates of AVS in a number of small retrospective studies, as incorrect catheter placement can be identified at time of procedure and corrected [12**–**16]. This reduces the reliance of radiological appearance and experience to determine catheter placement. As such may allow greater access to adrenalectomy for suitable patients in less experienced centres. At present the use of intraprocedural cortisol testing is not routine.

## Methods

Individuals undergoing AVS at the at the Royal Adelaide Hospital, South Australia from 2013 to 2021 were retrospectively reviewed. We compared the success rates of AVS before and after the introduction of intraprocedural cortisol testing using the QCA strip (Quick Cortisol Kit Q-CTZ-1000; Trust Medical Corporation) to inform on success of AV cannulation at the time of the procedure. All patients underwent a uniform diagnostic work up and had positive plasma aldosterone/renin ratio and seated saline suppression test in accordance with the Endocrine Society Clinical Practice Guideline [8]. A uniform AVS protocol was utilised and co-ordinated by a dedicated endocrine testing nurse. All AVS procedures were performed during continuous 50 μg/h Synacthen infusion (250 μg in 250 ml N saline, 50 ml per h) which was commenced 30 min before AVS and continued until AVS completion [8]. If QCA strip were available, they were used. Clinical information did not determine use, only availability. Procedures were predominantly performed by two consultant radiologists of similar experience, assisted by registrars (trainee radiologists).

### Primary outcome

The primary outcome was the success rates of the procedure which was defined by successful cannulation of both adrenal veins determined by formal cortisol concentration ratio between the adrenal vein and peripheral vein of >3 (post co-syntropin/Synacthen). This is consistent with AACB harmonisation guidelines used in Australia and the Endocrine Society’s Clinical Guideline [8].

### Secondary outcome

The secondary outcomes were (1) the number of samples taken, (2) theatre time used, (3) proportion of patients demonstrating lateralisation and the (4) number of patients referred to adrenalectomy with lateralisation.. In accordance with the Endocrine Society’s Clinical Guideline, AVS was considered to show lateralisation if the aldosterone/cortisol ratio in one adrenal vein was >4 fold that in the contralateral adrenal vein with >3 being borderline [8]. A cost analysis was performed based on cost of RCT ($40.00 AUD) testing kits used, the average number of blood collections in our data set, the cost of AVS in South Australia ($1500) and the number of times RCT had to be used to prevent a failed procedure (1—absolute risk reduction of non-diagnostic AVS). The effect of baseline success rate was modelled to assist various centres determine the impact of cost on that centre. The following formula was used:

$${{{{{{{\mathrm{Cost}}}}}}}} =	\; \left( {{{{{{{{\mathrm{RCT}}}}}}}}\;{{{{{{{\mathrm{strip}}}}}}}}\;{{{{{{{\mathrm{cost}}}}}}}}\; \times \;{{{{{{{\mathrm{samples}}}}}}}}\;{{{{{{{\mathrm{collected}}}}}}}}} \right) \\ 	 \, \times \left( {{{{{{{{\mathrm{NNT}}}}}}}}\;{{{{{{{\mathrm{to}}}}}}}}\;{{{{{{{\mathrm{prevent}}}}}}}}\;{{{{{{{\mathrm{non-diagnostic}}}}}}}}\;{{{{{{{\mathrm{test}}}}}}}}} \right)-{{{{{{{\mathrm{Cost}}}}}}}}\;{{{{{{{\mathrm{of}}}}}}}}\;{{{{{{{\mathrm{procedure}}}}}}}}{{{{{{{\mathrm{.}}}}}}}}$$

Statistical analysis was performed in the software package Prism (Version 9.4.0). Where data is normally distributed a non-paired two-sided test has been used, if normality has not been demonstrated Mann-Whitney test has been used. Fisher Exact test has been used for analysis of categorical variables and contingency tables.

## Results

**Fig. 1:**
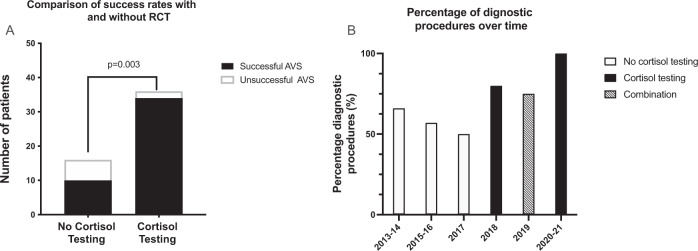
RCT and AVS success rate. The left panel demonstrates the number of successful (black) and unsuccessful (white) procedures with RCT and without RCT. The right panel demonstrates the success rates according to year, with RCT being introduced in 2018. The time without RCT is represented by white, with RCT black and combination of RCT and no RCT hashed.

**Fig. 2:**
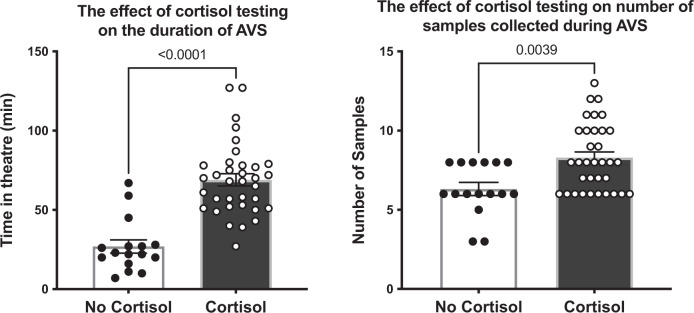
Effect of RCT on time in theatre and number of samples collected. The left panel represents the time spent in theatre in minutes. White with black circles demonstrates the procedures without RCT whilst black those with white circles RCT. The bar represents the mean time ± the SEM. Individual procedure times are represented by circles. The right panel depicts the number of samples taken during each AVS. White with black circles demonstrates the procedures without RCT whilst black with white circles those with RCT. The bar represents the mean time ± the SEM. The number of samples taken in each procedure are represented by circles. *P* values have been derived using an unpaired two sided *t*-test.

The baseline demographics in the RCT and non-RCT group were not significantly different (Table 1). Of note, the renin values tended to be lower in the RCT group at baseline. This likely reflects an increase in the functional sensitivity of the assay over time. The use of intraprocedural cortisol testing began in 2018 but was not uniformly performed and so for the 2019, procedures were performed with and without testing strips. 16 procedures were performed without RCT with a success rate of 62.5%, as compared to 37 procedures with RCT with a success rate of 94% (*p* = 0.003) (Fig. 1a). The success rate progressively increased over time following the introduction of RCT in 2018 (Fig. 2a). Four patients underwent a repeat AVS in the non-RCT group all were successful on second attempt. No patients from the RCT group underwent repeat.

**Table 1:** Demographics.

	No RCT	RCT	*P* value
Age (years)	51.6 ± 13.8	51.8 ± 12.1	0.94
Gender (M/F)	10/6	19/18	0.6
Systolic blood pressure (mmHg)	161 ± 27	164 ± 26	0.7
Number of AVS performed per year	4.3	9.8	0.06
Lateralisation	31%	49%	0.37
Right to Peripheral cortisol ratio	14.0 ± 8.0^a^	21.7 ± 14.1	0.1
Left to Peripheral cortisol ratio	11.5 ± 6.7^a^	13.3 ± 6.6	0.5
Dominant to non-dominant ratio of Aldosterone:Cortisol	10.6 ± 9.6	16.0 ± 21.8	0.44
Aldosterone pre saline suppression	768 ± 571	707 ± 429	0.68
Renin pre saline suppression (uIU/ml)	5.1 ± 4.1	3.3 ± 2.0	0.33
Aldosterone renin ratio pre saline suppression (pmol/L)	252 ± 291^b^	259 ± 183^b^	0.7
Aldosterone post saline suppression (pmol/L)	485 ± 467	438 ± 426	0.7
Aldosterone: renin ratio post saline suppression	144 ± 115^b^	250 ± 215^b^	0.7

^a^Data not available for five patients as aldosterone was not measure due to failed catheterisation.

^b^Large variation exists as unable to generate ratio if renin undetectable. In such cases renin assumed to be minimum detectable value.

**Fig. 3:**
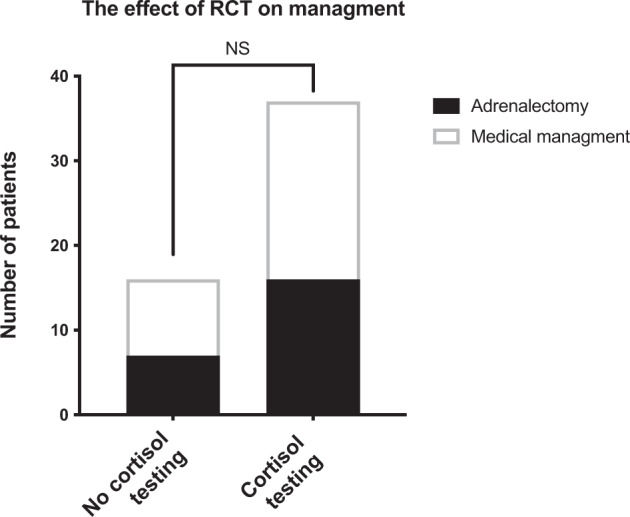
RCT and referral for adrenalectomy. Figure 3 represents the number of patients referred for adrenalectomy or chosen for medical management with and without RCT.

Procedural time was significantly higher with RCT (69 ± 23 vs 27 ± 17 min, *p* < 0.001) (Fig. 2A). The number of samples collected was significantly higher with RCT compared to no RCT (6 ± 1.6 vs 8 ± 2.1 samples, *p* = 0.004) (Fig. 2B). The absolute rates of referral for adrenalectomy were not significantly different between the RCT and no RCT group 39% vs 36%, respectively (Fig. 3). Of note, three of eight patients referred for adrenalectomy in the no RCT arm were referred despite non-diagnostic AVS.

**Fig. 4:**
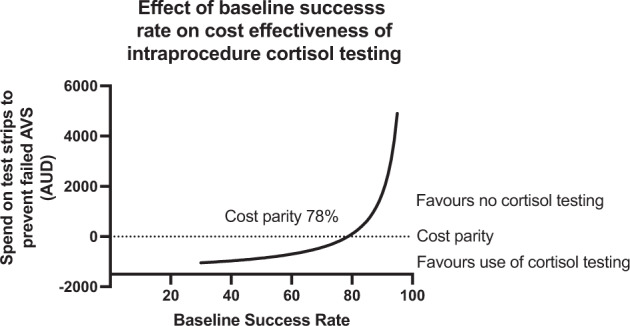
Effect of baseline success rate on cost effectiveness of intraprocedural cortisol testing. This a graphical representation of the cost in Australian Dollars of using RCT to prevent a single failed AVS. This analysis takes into account and the cost of the strips—the average number we of samples analysed (eight samples) if using RCT. It assumes an increase to a 100% success rate. It assumes a cost of $40 per strip.

Cost analysis demonstrated that, based on the observed improvement in success rate, we saved an average of $103 per AVS and that it would remain financially beneficial to use RCT during AVS until the pre RCT test rate reached 78% (Fig. 4).

## Discussion

In this study we examined the impact of RCT on AVS outcomes, the impact on referral for adrenalectomy and the cost of using this intervention. We analysed retrospective AVS data from the Royal Adelaide Hospital between the years of 2013–2021. We demonstrate that the use of RCT was associated with an increase in the diagnostic success rate of AVS from 62% to 94%. This change occurred rapidly after the introduction of RCT. Interestingly, examination of secular success rate reveals a drop-in success rate in 2019 when RCT was not available for the entire 12-month period and thus was used in only 75% of procedures (versus 80% for 2018) and then increasing back to 100% after RCT availability resumed.

This increase in success rate associated with RCT was accompanied by an increased number of samples collected and time spent in theatre compared to without RCT. It is likely that this reflects the identification of incorrect catheter placement and need to reposition and collect additional samples with real time feedback from the RCT. The initial logistical set up at our institution required samples to transported to the central laboratory 1 floor below and then centrifuged. This likely explains the substantially increased mean theatre time with RCT of 69 vs 27 min without RCT (*P* < 0.01). We have subsequently amended the protocol to use an in-suite centrifuge with marked reduction in theatre time (data not presented).

We generated a cost analysis based on the pre-RCT success rate and demonstrate that based on the improvement seen in our centre, the cost of RCT and the cost of repeat procedures that it was likely to be financially beneficial if the pre-RCT success rate is less than 78%. This model is simple and limited. It likely underestimated the cost effectiveness of RCT. It does not take into account cost of recurrent failed procedures (i.e. repeating AVS 2 times or more), patient cost or increased morbidity (particularly due to prolonged alternative anti-hypertensive therapy and hypokalaemia which are not insignificant).

This study reports similar results to prior analysis of the effect of intraprocedural cortisol testing and adds weight to the standardised use of RCT for AVS [12**–**16]. These studies demonstrate and average success rate of 69% (range 54–90%) before introduction of RCT and a success rate of 89% (range 80–97%) after the introduction. The rate of successful procedures pre RCT varies significantly, ranging from 44 to 96% (supplementary table 1) [14, 17–24]. Unlike other studies, this study reports on referral rates for adrenalectomy as an outcome, although it was under powered to detect a significant difference. It also provides a financial analysis to assist centres in deciding if implementing RCT to AVS programs is financially viable. Like previous studies, this study is limited by its retrospective nature and potential risk of bias—particularly that of improvement over time. However, whilst we acknowledge that more procedures were performed per year whilst RCT was being used, this is mitigated by the fact there is temporal overlap of the two techniques and rapid increase success rates after the introduction of RCT as well as the decrease in success rate when it was not available. An additional limitation is inability to comment on the long-term consequence of RCT use on blood pressure and biochemical markers.

## Conclusion

Intraprocedural cortisol testing improved the success rates of AVS in a medium throughput centre to that comparable of the most successful centres by allowing identification of failed cannulation of the adrenal vein and will likely facilitate less experienced centres to offer this important diagnostic service. Additionally, we have demonstrated that this will be cost effective if the pre-RCT success rate is less than 78%.

### Summary

#### What is known about topic?

- Primary hyperaldosteronism is the most common cause of secondary hypertension.
- Identification of unilateral secretion is required for surgical resection.
- Surgical resection is superior to medical management.
- AVS is the gold standard for identifying unilateral secretion.
- AVS has high failure rates, this may impact ability to refer for resection.
- Real time cortisol testing (RCT) has been suggested to improve accuracy.

#### What this study adds?

- Real world data confirming the impact RCT on AVS success rates in a medium throughput centre.
- Practical implications for setup—effect on number of samples collected and time in theatre.
- Cost analysis of implementing real time cortisol measurement.
- The impact of RCT on referral patterns for adrenalectomy.

## Supplementary information

Supplemental Table

## Data Availability

Data are included within the paper, request for additional data can be directed to the corresponding author.
