# Supplementary material for: Intraprocedural cortisol testing improves adrenal vein cannulation success and diagnostic accuracy in assessment of primary aldosteronism, in a medium throughput centre
Source: J Hum Hypertens. 2022 Sep 30;37(9):783–7. doi: 10.1038/s41371-022-00756-z (PMC10471492; doi:10.1038/s41371-022-00756-z)
Supplement: Supplementary file 1 — Supplemental Table [file 41371_2022_756_MOESM1_ESM.docx]

| **Study** | **Number of participants** | **Success rate** | **Failed procedures attributed to failure of right or both adrenal veins** |
| --- | --- | --- | --- |
| Vonend 2011(1) | 306 | 41% | 83% |
| Deipolyi 2015(2) | 92 | 63% | 94% |
| Teng 2015(3) | 47 | 55% | Not reported |
| Dekkers 2016(4) | 96 | 96% | 100% |
| Ota 2016(5) | 125 | 99% | 100% |
| Page 2018(6) | 147 | 71% | 81% |
| Rossi 2011(7) | 1625 | 80% | Not reported |
| Lee 2020(8) | 48 | 90% | 60% |
| Kocjan 2020(9) | 259 | 77% | 79% |

**Supplemental table 1.**

References:

1. Vonend O, Ockenfels N, Gao X, Allolio B, Lang K, Mai K*, et al.* Adrenal venous sampling: Evaluation of the german conn's registry. Hypertension. 2011;57(5):990-5.

2. Deipolyi AR, Bailin A, Wicky S, Alansari S, Oklu R. Adrenal vein sampling for conn's syndrome: Diagnosis and clinical outcomes. Diagnostics (Basel). 2015;5(2):254-71.

3. Teng J, Hutchinson ME, Doery JC, Choy KW, Chong W, Fuller PJ*, et al.* Role of adrenal vein sampling in primary aldosteronism: The monash health experience. Intern Med J. 2015;45(11):1141-6.

4. Dekkers T, Prejbisz A, Kool LJS, Groenewoud H, Velema M, Spiering W*, et al.* Adrenal vein sampling versus ct scan to determine treatment in primary aldosteronism: An outcome-based randomised diagnostic trial. The lancet Diabetes & endocrinology. 2016;4(9):739-46.

5. Ota H, Seiji K, Kawabata M, Satani N, Omata K, Ono Y*, et al.* Dynamic multidetector ct and non-contrast-enhanced mr for right adrenal vein imaging: Comparison with catheter venography in adrenal venous sampling. Eur Radiol. 2016;26(3):622-30.

6. Page MM, Taranto M, Ramsay D, van Schie G, Glendenning P, Gillett MJ*, et al.* Improved technical success and radiation safety of adrenal vein sampling using rapid, semi-quantitative point-of-care cortisol measurement. Ann Clin Biochem. 2018;55(5):588-92.

7. Rossi E, Regolisti G, Perazzoli F, Negro A, Grasselli C, Santi R*, et al.* Intraprocedural cortisol measurement increases adrenal vein sampling success rate in primary aldosteronism. Am J Hypertens. 2011;24(12):1280-5.

8. Lee J, Kang B, Ha J, Kim MH, Choi B, Hong TH*, et al.* Clinical outcomes of primary aldosteronism based on lateralization index and contralateral suppression index after adrenal venous sampling in real-world practice: A retrospective cohort study. BMC endocrine disorders. 2020;20(1):114.

9. Kocjan T, Jensterle M, Vidmar G, Vrckovnik R, Berden P, Stankovic M. Adrenal vein sampling for primary aldosteronism: A 15-year national referral center experience. Radiol Oncol. 2020;54(4):409-18.
